# Into the realm of social capital for adolescents: A latent profile analysis

**DOI:** 10.1371/journal.pone.0212564

**Published:** 2019-02-21

**Authors:** Mikael G. Ahlborg, Petra Svedberg, Maria Nyholm, Antony Morgan, Jens M. Nygren

**Affiliations:** 1 School of Health and Welfare, Halmstad University, Halmstad, Sweden; 2 Glasgow Caledonian University in London, London, United Kingdom; University of Pennsylvania, UNITED STATES

## Abstract

**Background:**

Recent reports of increasing prevalence of frequent health complaints and mental health problems among adolescents call for directing more attention on determinants of adolescent health. The relationship between health and social capital has gained increased attention since the early 2000’s and research at review level confirms the importance of social capital for health outcomes, despite methodological heterogeneity. The aim of this study was to identify distinct profiles of family, school and peer social capital in a nationally representative sample of adolescents and to explore health outcomes in those profiles.

**Method:**

Cross-sectional data from the Swedish Health Behaviour of School-aged Children 2013/14 was used for this study. The analytical sample consisted of 7,804 adolescents aged 11-, 13- and 15-years. Items representing sense of belonging and emotional support were assessed in three contexts; family, school and among peers. Latent profile analyses (LPA) were run to determine social capital profiles. Health outcomes included frequent health complaints and life satisfaction, while socioeconomic status and genders were included as predictors.

**Results:**

The results show that five distinct profiles best represent the data for 11- and 15-year olds, while a four-profile model was optimal for 13-year olds. Some profiles were recurrent between age groups but unique profiles were also found. Health outcomes were significantly different between profiles depending on levels of social capital in the different contexts.

**Conclusions:**

This study provides novel insight into how social capital co-occurs among adolescents within the contexts of family, school and peers and how this translates into differences in health outcomes. The national representativeness of the sample increases the implications of the results and contributes to meaningful insights that help explain the interactions of social capital in multiple contexts, complementing what is previously known about the relationship with adolescent health.

## Introduction

Investment in adolescent health today will undoubtedly have benefits in decades to come. This arises from evidence that demonstrates that health inequalities established in childhood and young adulthood are likely to persist into adulthood [1]. Consequentially, the case for directing more attention to the determinants of adolescent health and well-being is strengthened. Given, that the prevalence of mental health problems and self-rated health complaints is increasing among adolescents in many high-income countries, it can be argued that particular attention should be placed on preventive strategies for improvements in these types of outcomes [2–4]. In Sweden, a recent report focussing on trends in adolescent health, reported a general increase in mental health problems, psychiatric diagnoses and prescribed medication for treatment of mental health problems over the last decade [5]. Meanwhile, issues concerning equality persist since socioeconomic inequalities in adolescent health have been increasing over the last decade [6]. As such, the discourse around where and how resources should be distributed to maximize efficiency of preventive and therapeutic strategies has grown [1]. Although socioeconomic status (SES) has been established as an important component in the explanation of health inequalities among adolescents [7, 8], the interest in protective factors have led researchers to move beyond SES and towards research that encompasses related factors that can either buffer, moderate or mediate, its impact on health. The concept of social capital is one example of a protective factor that has received increasing attention since the early 2000’s. It is argued to contribute to improved understandings of how psychosocial pathways can help to minimise socioeconomic health inequalities [9].

### Social capital

Social capital in a broad sense refers to the notion that social networks act as a potential resource for individuals and society as a whole [10]. The concept has been extensively discussed by Bourdieu [11] in the mid 1980s, followed by Coleman [12], Putnam [13] and more recently Kawachi and colleagues [14]. Together, these authors have contributed to differing but overlapping understandings of how social networks can provide value to health enhancement. Over the last few decades a multitude of research has arisen that provides a nuanced debate about its definition and measurement [15–17]. At present, two typologies of social capital distinguish between structural and cognitive phenomenon [18]. Structural social capital involves more tangible measures (network size etc.) while cognitive social capital is based on subjective appraisals of social relations. Assessments of social capital in the adult population has relied much on measuring connectedness, participation, feelings of trust and experienced norms within communities/neighbourhoods, organisations and social networks [18]. Approaches to assess social capital among adolescents have had great similarities with those used for adult populations, taking account of contexts extending from neighbourhood to family, schools and peers. The work of Morrow [19, 20] helped to elaborate social capital in relation to children and adolescents. Although she recognised that there was not doubt about its usefulness in health-related research, its constraints had to be acknowledged. Attention needed to be paid to existing conceptual differences, varying measurement methods and the gap between adolescent and adult perceptions of for example ‘societal participation’. As a result, more recently researchers have been advised to be more precise and make explicit the particular social capital perspective they are investigating, acknowledge its complexities and interpretations and their rationale for the ways they are measuring it [21, 22]. Simply transferring social capital, a construct that has been principally discussed within an adult framework, to research on adolescents can thus generate problems associated with conceptualisation and methodological validity [23, 24]. Therefore, tools to investigate the relevance of social capital to young people’s health and wellbeing should be developed specifically in the context of adolescents’ life worlds [24]. The current study focuses on two constructs of cognitive social capital, namely social cohesion and social support, and directs particular attention to perceptions of social relations regarding sense of belonging and emotional support. The operationalization of sense of belonging and emotional support as indicators of social capital used here is drawn from the work of Alvarez and colleagues [25, 26]. Among adolescents, the contexts of family, school and peers have previously been described as key settings within which social capital can generate health benefits [19]. The Health Behaviour of School-aged Children (HBSC) network, concur from a methodological standpoint that these contexts are crucial in research that aims to identify risks of and resilience against poor health [27].

The relationship between social capital and various health outcomes has been extensively investigated in adult populations [28–33]. Additional research provides evidence that social capital can have a buffering effect on the impacts of SES on health [9]. Even though there is less research available on this relationship within the context of adolescent health, there is clear evidence of a linear relationship between social capital and adolescent health that shows how stronger social capital is associated with improved health [34, 35]. Furthermore, review level research indicate that social capital is important for adolescent health outcomes, even though the evidence is drawn from a heterogeneous (in terms of dimensions of social capital and contexts studied) study base [36]. The most common contexts investigated in social capital research on adolescents are family, school, neighbourhood, and peer relations. However, social media and increased internet-use has created new opportunities for the creation of social capital. Whilst social media as a setting is outside the scope of this study, important questions arise. For example: what significance does social media have for the formation of social capital; is their impact already integrated into established contexts or is their impact independent of these and therefore worthy of independent study. The sub-concept of family social capital, originally introduced by Coleman, is today described in the context of the wider family—extending beyond the traditional parent-child relationship to include siblings and other family members [26]. Multiple dimensions of family social capital such as social interaction with family members (activities), sense of belonging (respect, trust, closeness) and emotional support (openness to confide in family members, empathy, love) have shown to be significantly associated with the health of adolescents [26]. Similar findings have been found in other studies on neighbourhood and school social capital, indicating both an independent relationship [34] but also, in combination with family social capital, an additive association with adolescent health [37]. In addition to these results, others have found support for peer social capital as a determinant of adolescent mental health [38] and a linkage to risk behaviours depending on the norms of the peer group [39].

### Rationale

Apart from establishing a relationship between social capital and adolescent health, previous research has identified multiple contexts in which social capital exists and operates as a source of value for individuals. Variable focussed analysis, such as regression analysis, can tell us about to what degree social capital in different contexts can predict health outcomes and help us explore the form of these relationships. Although this has great value for the understanding of social capital and adolescent health, there are other research questions that require a more exploratory approach. To be able to investigate if there are certain patterns or combinations of social capital among adolescents that generally co-occur and how these translate into differences in health outcomes, a person-centred approach might be useful. As such, Latent Profile Analysis (LPA) is of an exploratory nature and ensues a person-centred approach to uncover subgroups and explore patterns within populations [40]. By identifying latent profiles, this type of analysis has been suggested to help unravel some of the complex interactions that occur between independent variables that may, to a varying degree, be associated with one another [41]. Latent profiles can then be used to provide insights into the association with dependent variables. We are unaware of other studies that have applied this kind of analysis to research on social capital in relation to adolescent health. The aim of this study was to add to existing research by identifying social capital profiles through LPA and using these to explore health outcomes.

## Methods

### Data

This study uses data from the World Health Organisation [42] Health Behaviour in School Aged Children (HBSC) survey, which collects data every four years from more than 40 countries in Europe and in North America. The survey collects data from nationally representative samples within each country and uses self-reporting questionnaires, administered to classes by teachers. Participation in the survey is voluntary and implicit consent is sought from the parents prior to the administering of the questionnaire, Questionnaires are only assigned a consecutive identification number and therefore remain anonymous. Information about the HBSC survey, data management and confidentiality is given to parents and adolescents before the survey is conducted. A two-stage cluster sampling procedure is conducted to obtain a nationally representative sample. Three age groups are included, 11-, 13- and 15-year olds. The present study is based on data from the Swedish HBSC-survey conducted in 2013/14. Data were accessed upon request from the Public Health Agency in Sweden. There were 11,328 eligible respondents in all randomly selected classes out of which 7,867 participated in the survey, which gives a response rate of 69,4 per cent [43]. The analytical sample in this study consisted of 7,804 adolescents since 63 adolescents failed to respond on the items chosen for this particular study. Respondents were distributed equally between gender and age. The study design was approved by the Regional ethical review board at Lund University (ref nr HH F2018/30).

### Variables

#### Social capital variables

Social capital was measured in this study using items reflecting the constructs of social cohesion (trust and sense of belonging) and social support within three contexts; family social capital (four items), school social capital (seven items) and peer social capital (four items). These measures were included in the 2013/14Swedish HBSC-questionnaire [43]. Family social capital comprised four items (Cronbach’s alpha = 0,84), all beginning with ‘in my family we…’ followed by: ‘talk about important things’, ‘listen to each other’, ‘ ask when we don’t understand’ and ‘resolve misunderstandings’. Response alternatives ranged between ‘strongly disagree’ (1) to ‘strongly agree’ (5). The face validity of these four items have been strongly supported in adolescent focus groups internationally, labelling them as ‘the reality of family life’ [27]. School social capital was measured with seven items (Cronbach’s alpha = 0,83). First, participants answered the question ‘How do you feel about school at present?’ rated as ‘I don´t like at all’ (1) through to ‘I like it a lot’ (4). Secondly, participants were asked to assess their relationships with classmates using three items: ‘students enjoy being together’, ‘most students are kind and helpful’ and ‘students accept me as I am’. Similarly, teacher-student relations were assessed using the following three items: ‘teachers accept me as I am’, ‘teachers care about me’, ‘I feel a lot of trust in my teachers’. Response alternatives of these six items ranged from ‘strongly disagree’ (1) to ‘strongly agree’ (5). Lastly, a four-item measure assessed peer social capital (Cronbach’s alpa = 0,94). ‘My friends really try to help me’, ‘I can count on my friends when things go wrong’, ‘I have friends I can share my joys and sorrows’ and ‘I can talk about my problems with my friends’. Response alternatives ranged from ‘very strongly disagree’ (1) to ‘very strongly agree’ (7). A summarized score was calculated for each context of social capital resulting in the following ranges; 4–20 for family social capital, 7–34 for school and 4–28 for peer social capital.

#### Outcomes and predictors

Health was assessed by two measures from the HBSC questionnaire, the HBSC-Symptom Checklist (SCL) and Cantril’s Ladder. The Symptom Check-List (SCL) is a bi-dimensional measure consisting of eight health complaints, four of somatic origin and four psychological. Respondents are asked how frequently during the last six months they have experienced the following; abdominal pain, backache, headache, dizziness (somatic), feeling low, irritable, anxious, trouble sleeping (psychological). Response alternatives range from ‘seldom or never’ (1) to ‘almost every day’ (5). In this study we utilized a threshold to assess how many complaints respondents’ experienced equal to or above ‘more than once a week’ (score 4 or above), resulting in either a 0 or 1 for each symptom (total range 0–8). This threshold has previously been used as an indicator of frequent health complaints that may impair the function in everyday life [44]. Cantril’s Ladder is a measure of life satisfaction where respondents are asked to rate how they feel about their life in general at the moment, using a picture of a ladder. The bottom of the ladder represents the worst possible (0) life and the top the best life possible (10), giving a response range of 0–10. Included as a predictor, socioeconomic status was assessed via the Family Affluence Scale (FAS), a self-report measure derived from items included in the HBSC questionnaire. FAS consists of six items reflecting material wealth, such as car ownership and frequency of travelling abroad [45]. A summarized score of FAS was calculated with a range of 0–13 with higher values indicating high socioeconomic status. Gender was also included as a predictor and an evaluating variable to explore potential differences in the profile characteristics.

### Statistical analysis

Descriptive analysis: Three data sets, one for each age group (11, 13 and 15), were constructed before latent profile analysis was conducted and descriptive analyses were produced using SPSS 21.0 (IBM). First, mean values with standard deviation were calculated for social capital dimensions in each age group and for boys and girls separately. Pearson’s correlation coefficient was used between the three social capital variables in each age group to denote the strength of the linear association between social capital variables.

Latent profile analysis: Latent profile analysis (LPA) was used with the purpose of uncovering underlying subgroups (i.e. profiles) within the sample. LPA is a mix modelling technique that aims to build typologies based on individual responses from a set of observed variables [46], which in our study were represented by social capital in three different contexts. LPA was run for each dataset respectively using Mplus Software v.8. We estimated and compared LPA models with an incremental number of profiles to identify the best model fit. Models of up to eight profiles were tested in this study. Following the procedure suggested by Tein, Coxe and Cham [46], a number of model fit criteria were considered when comparing models to obtain the best solution; Log-likelihood (LL), Bayesian information criteria (BIC), Sample-size adjusted BIC (SSA-BIC), Entropy, Lo-Mendell-Rubin adjusted likelihood ratio test (LMR) and Bootstrapped likelihood ratio test, (BLRT). Lower values of LL, BIC, SSA-BIC indicate better model fit. Entropy is a measure of aggregated classification uncertainty with values closer to 1 indicating a more certain separation between profiles. LMR and BLRT show how well the number of profiles fit the data compared to a solution with one less profile (K-1). The two tests both provide a p-value that indicates if the current number of profiles fit the data better than K-1 (p<0.05). Those models that indicated a good fit to the data were investigated more closely regarding their composition, profile sizes preferably above 1% [47], and interpretability (standardized scores for each social capital profile). To examine a possible clustering effect with reference to school classes in the sample, a two-level mixture modelling technique was applied for each age group. If an effect was detected, the corrected model was acknowledged and included in the results.

Distal outcomes: Health variables, frequent health complaints and life satisfaction, along with FAS and gender were included in LPA as auxiliary variables (not affecting the models) to produce distal (evaluating) outcomes for each profile. Profiles were compared pairwise within each age group to explore differences in distal outcomes means and probabilities between profiles (p-value to indicate significant difference was set at *p*<0.05).

Multiple regression analysis: To see what information LPA might add to the linear relationship between social capital and adolescent health as expressed through regression analysis, multiple regression analysis was conducted in SPSS 21.0 (IBM). Social capital variables were treated as independents and health outcomes as dependent variables. Models were adjusted for gender and SES. Latent profiles were then added and model fit indices, Akaike’s information criteria (AIC) and an F-test, were used to compare models.

## Results

### Descriptive statistics

Descriptive statistics and correlations between family, school and peer social capital for each age group are presented in Table 1. Lower mean values were observed for older adolescents than for younger. Pearson’s correlation showed a stronger linear relationship between family and school social capital than of family-peer and school-peer in all age groups, with 34–39 per cent of the variance explained across age groups. As displayed in Table 1, there were significant gender differences in social capital. The largest difference was found in peer social capital where girls had a mean score that is around two points higher than boys in all age groups. However, no significant difference between boys and girls was found in family social capital among 13- and 15-year olds.

**Table 1:** Mean values, standard deviation and correlation between social capital variables in all age groups and between boys and girls.

	Total				Boys		Girls		diff.
	Mean	SD	1	2	Mean	SD	Mean	SD	*p*
**11-year olds n = 2677**									
Family social capital (4–20)	17.49	2.40	**-**		17.39	2.50	17.61	2.29	0.02*
School social capital (7–34)	29.57	3.76	**0.39**	**-**	29.30	3.98	29.83	3.52	<0.01*
Peer social capital (4–28)	22.76	6.16	**0.30**	**0.35**	21.95	6.23	23.58	5.96	<0.01*
**13-year olds n = 2306**									
Family social capital (4–20)	17.03	2.67	**-**		17.09	2.62	16.97	2.72	0.29
School social capital (7–34)	28.12	4.16	**0.39**	**-**	28.35	4.09	27.91	4.18	0.01*
Peer social capital (4–28)	22.71	6.18	**0.24**	**0.25**	21.56	6.39	23.79	5.82	<0.01*
**15-year olds n = 2823**									
Family social capital (4–20)	16.73	2.88	**-**		16.68	2.88	16.81	2.88	0.21
School social capital (7–34)	27.10	4.24	**0.34**	**-**	27.49	4.32	26.75	4.12	<0.01*
Peer social capital (4–28)	22.33	5.95	**0.26**	**0.27**	21.38	6.05	23.26	5.72	<0.01*

1, Family social capital;

2, School social capital.

Pearson correlation coefficient, values in bold indicate *p*<0.01.

Independent t-tests,

* = *p*<0.05 (sig. 2-tailed).

diff., difference between boys and girls.

### Determining the optimal number of profiles

The procedure for identifying the optimal number of profiles was done independently for 11-, 13- and 15-year olds. The model fit criteria values are shown in Table 2. All models that were under consideration were investigated more closely regarding their composition (profile sizes >1%), interpretability and class probability (most likely latent class membership >0.80). Altogether this investigation and the model fit criteria combined, pointed to a 5-profile model for 11-year olds and a 4-profile model for 13-year olds. For 15-year olds, while LMR favoured a 3-profile model, consideration of other model fit criteria combined with consideration of theoretical meaningfulness and subjective dimensions of choice pointed to a 5-profile model being viewed as optimal.

**Table 2:** Goodness of fit indices, entropy and model comparisons for latent profile models in all age groups.

Model	LL	BIC	SSA-BIC	Entropy	LMR	BLRT
**11-year olds**						
1 profile	—10776.838	21601.03	21581.97			
2 profile	—10097.573	20274.07	20242.30	0.87	<0.01	<0.01
3 profile	—9871.820	19854.13	19809.65	0.84	<0.01	<0.01
4 profile	—9676.716	19495.43	19438.30	0.85	<0.01	<0.01
**5 profile**	**—9554.951**	**19283.54**	**19213.64**	**0.86**	**<0.01**	**<0.01**
6 profile	—9436.921	19079.05	18996.44	0.86	>0.05	<0.01
7 profile	—9359.702	18956.18	18860.86	0.87	<0.05	<0.01
8 profile	—9212.724	18693.79	18585.76	0.89	>0.05	<0.01
**13-year olds**						
1 profile	—9423.17	18892.80	18873.74			
2 profile	—8943.79	17965.02	17933.25	0.86	<0.01	<0.01
3 profile	—8690.99	17490.39	17445.91	0.87	<0.01	<0.01
**4 profile**	**—8560.45**	**17260.27**	**17203.08**	**0.87**	**0.05**	**<0.01**
5 profile	—8475.33	17121.02	17051.12	0.87	>0.05	<0.01
6 profile	—8419.59	17040.50	16957.90	0.86	>0.05	<0.01
7 profile	—8359.59	16951.48	16856.16	0.86	>0.05	<0.01
8 profile	—8253.93	16771.12	16663.10	0.86	>0.05	>0.05
**15-year olds**						
1 profile	—11541.65	23130.97	23111.90			
2 profile	—11042.62	22164.70	22132.92	0.84	<0.01	<0.01
3 profile	—10833.06	21777.35	21732.87	0.84	<0.01	<0.01
4 profile	—10720.67	21584.35	21527.16	0.85	>0.05	<0.01
**5 profile**	**—10596.32**	**21367.45**	**21297.55**	**0.85**	**<0.05**	**<0.01**
6 profile	—10512.69	21231.96	21149.34	0.85	>0.05	<0.01
7 profile	—10454.34	21147.05	21051.73	0.86	>0.05	<0.01
8 profile	—10421.05	21112.25	21004.22	0.86	>0.05	<0.01

LL, Log-Likelihood; BIC, Baeysian Information Criterion; SSA-BIC, Sample-Size Adjusted BIC; LMR, Lo Mendell-Rubin test; BLRT, Bootstrapped Likelihood Ratio Test.

Optimal model within each group are in bold and highlighted in green.

### Social capital latent profiles

**Fig 1:**
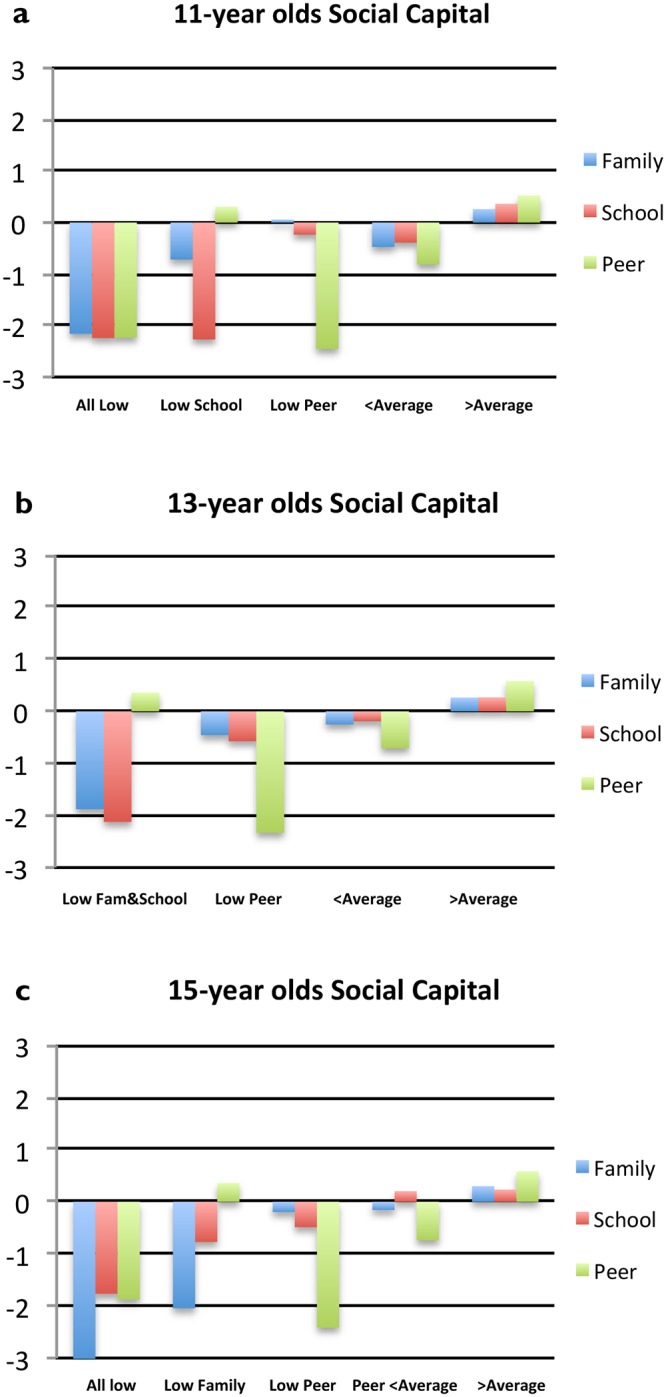
a-c. Standardized scores of family, school and peer social capital for optimal latent profile analysis model in each age group.

The standardized scores (z) for the different profiles for 11–13- and 15-year olds are graphically illustrated in Fig 1a–1c. Due to the scores being standardized, the average score for the entire sample within that age group is equal to zero (0) in the figures, meaning negative scores indicate that individuals scored lower than average and positive scores above average. Each profile is given a label according to the typology of the profile (Fig 1a–1c).

Profiles for 11-year olds (Fig 1a): As the two-level mixture analysis revealed a class clustering effect for this group, it is the corrected model that is presented for. Profile 1 is characterised by low scores in all social capital contexts, between z = -2.16 and z = -2.24. Profile 2 is characterised low school social capital (z = -2.27), below average family social capital (z = -0.72) and above average peer social capital (z = 0.31). Profile 3 includes individuals with very low peer social capital (z = -2.46) while average scores in family and school social capital. Profiles 4 and 5 are not characterized by a social capital variable standing out significantly from the others but rather that social capital scores in the three contexts are slightly below average in profile 4 and above average in profile 5.

Profiles for 13-year olds (Fig 1b): Here, a 4-profile model was considered optimal. In Profile 1, individuals score very low on family (z = -1.88) and school social capital (z = -2.12), while peer social capital is slightly above average. In contrast, profile 2 is characterized by a very low score on peer social capital (z = -2.33). Profile 3 and 4 are mirroring slightly below and above the average scores with the difference in peer social capital scores being greater between the two profiles than the scores in family and school social capital.

Profiles for 15-year olds (Fig 1c): The model including five profiles was deemed optimal. Individuals in profile 1 are characterized by scoring very low on family social capital (z = -3.11) but also considerably below average on school (z = -1.76) and peer social capital (z = -1.88). Profile 2 shares a low score on family social capital (z = -2.04) while school social capital is closer to average and peer social capital above average (z = 0.36). In contrast, profile 3 is characterized by very low scores on peer social capital (z = -2.41) with scores closer to average on family (z = -0.20) and school social capital (z = -0.49). In profile 4, peer social capital is scored below average (z = -0.74) with scores of family and school social capital close to average. Lastly, individuals characterized by above average scores on all three contexts of social capital are found in profile 5.

### Differences in outcomes between profiles

Differences in health complaints, life satisfaction, family affluence and genders between profiles are presented in Table 3.

**Table 3:** Differences in health outcomes, family affluence and gender distribution between latent profiles in each age group.

		Profile 1	Profile 2	Profile 3	Profile 4	Profile 5	Total
		Mean (S.E.)	Mean (S.E.)	Mean (S.E.)	Mean (S.E.)	Mean (S.E.)	Mean (S.E.)
**11-year olds**	*Label*	*All Low'*	*Low School'*	*Low Peer'*	*<Average'*	*>Average'*	
		n = 59	n = 106	n = 157	n = 512	n = 1843	n = 2677
*Health complaints (0–8)*		2.91^a^ (0.30)	2.74^a^ (0.22)	1.37^b^ (0.14)	1.24^b^ (0.07)	0.84^c^ (0.03)	1.07 (0.03)
*Life satisfaction (0–10)*		5.79^a^ (0.35)	6.49^a^ (0.24)	7.76^b^ (0.16)	7.37^b^ (0.08)	8.39^c^ (0.04)	8.02 (0.04)
*Family affluence (0–13)*		8.71^a^ (0.29)	9.09^a^ (0.23)	9.11^a^ (0.16)	8.97^a^ (0.09)	9.16^a^ (0.05)	9.11 (0.04)
*Boys/Girls (%)*		54/46	60/40	61/39	60/40	46/54	51/49
**13-year olds**	*Label*	*Low Fam*.*&School'*	*Low Peer'*	*<Average'*	*>Average'*		
		n = 71	n = 213	n = 502	n = 1520		n = 2306
*Health complaints (0–8)*		4.27^a^ (0.33)	2.19^b^ (0.17)	1.55^c^ (0.10)	1.33^c^ (0.05)		1.57 (0.04)
*Life satisfaction (0–10)*		3.89^a^ (0.35)	6.36^b^ (0.17)	6.99^c^ (0.09)	7.61^d^ (0.05)		7.21 (0.04)
*Family affluence (0–13)*		8.63^a^ (0.39)	8.69^a^ (0.16)	9.04^a^ (0.09)	9.37^b^ (0.05)		9.19 (0.04)
*Boys/Girls (%)*		30/70	59/41	69/31	42/58		50/50
**15-year olds**	*Label*	*All Low'*	*Low Family'*	*Low Peer'*	*Peer <Average'*	*>Average'*	
		n = 52	n = 96	n = 191	n = 680	n = 1804	n = 2823
*Health complaints (0–8)*		4.08^a^ (0.46)	3.86^a^ (0.33)	2.69^b^ (0.19)	1.81^c^ (0.10)	1.64^c^ (0.05)	1.89 (0.04)
*Life satisfaction (0–10)*		4.16^a^ (0.46)	4.63^a^ (0.29)	6.02^b^ (0.19)	6.50^c^ (0.08)	7.26^d^ (0.04)	6.83 (0.04)
*Family affluence (0–13)*		8.21^a^^,^^b^ (0.48)	8.44^a^ (0.30)	8.56^a^ (0.19)	9.06^b^ (0.09)	9.41^c^ (0.05)	9.20 (0.04)
*Boys/Girls (%)*		65/35	33/67	55/45	65/35	43/57	50/50

^a,b,c,d^ = Values in the same row that do not share a common subscript are significantly different at *p* < 0.05 (Total not included).

S.E. = Standard Error

#### Health outcomes

Health outcomes included self-reported frequent health complaints and life satisfaction. The profiles among 11-year olds that displayed the worst health outcomes were the ‘All Low’ and the ‘Low School’ profile (Table 3). Despite their differences in social capital levels there were no significant differences between the two in either health complaints or life satisfaction. Health outcomes were however significantly different from the other profiles. Health outcomes among individuals belonging to the ‘Low Peer’ profile were not significantly different from those of the ‘<Average’ profile despite their very poor levels of peer social capital. The ‘>Average’ profile included the lowest ratings of health complaints and the highest life satisfaction, significantly different from the other profiles. Among the profiles amid 13-year olds, individuals in the ‘Low Fam&School’ profile had very poor health outcomes, significantly different from the other profiles (Table 3). Individuals fitting the ‘Low Peer’ profile did not display as poor health outcomes, but still significantly different from the ‘<Average’ and ‘>Average’ profile. In 15-year olds, a profile labelled ‘All Low’ reappeared, with individuals that also rated their health as poor, however not significantly different from the ‘Low Family’ profile. Despite having peer social capital above average, individuals in the ‘Low Family’ profile had health outcomes very close to the ‘All Low’ profile. A profile in contrast to the ‘Low Family’, the ‘Low Peer’ displayed scores on health outcomes that were significantly better than the two previously mentioned, but also significantly worse than the ‘Peer <Average’ and the ‘>Average’ profile.

#### Socioeconomic status and genders

Socioeconomic status was assessed by FAS. Differences in mean scores between profiles were visible but few differences were significant (Table 3). Regarding 11-year olds no significant differences were found for family affluence between profiles. Among 13- and 15-year olds, the ‘>Average’ profiles had significantly higher scores than the other profiles. Concerning gender differences in social capital profiles, boys were clearly overrepresented in profiles with below average scores (Table 3). Only in the ‘Low Fam&School’ (13-year olds) and the ‘Low Family’ (15-year olds) profiles, girls accounted for the majority by about a two to one ratio. Instead, all ‘>Average’ profiles had a majority of girls.

#### Multiple regression results

Multiple regression analysis was conducted to show to which degree our social capital variables predict health outcomes in each age group respectively (Table 4). By adding the identified latent profiles into the second model only minor changes to the predictions of family, school and peer social capital on health outcomes were found. Thus, associations appear to be robust. Also, model fit indices (AIC and F-test) favour some of the models where latent profiles were added, however no specific pattern was detected.

**Table 4:** Multiple regression for health outcomes by social capital with model fit indices.

	Model 1			Model 2^a^			
	β	p	AIC	β	p	AIC	F-test
**11-year olds**							
**Health Complaints**							
Family Social Capital (4–20)	—0.08	<0.01	1501.54	—0.08	<0.01	1484.40	<0.01
School Social capital (7–34)	—0.11	<0.01		—0.08	<0.01		
Peer social capital (4–28)	—0.01	0.03		—0.01	0.53		
**Life satisfaction**							
Family Social Capital (4–20)	0.17	<0.01	1685.96	0.17	<0.01	1689.77	0.37
School Social capital (7–34)	0.15	<0.01		0.15	<0.01		
Peer social capital (4–28)	0.02	<0.01		0.03	<0.01		
**13-year olds**							
**Health Complaints**							
Family Social Capital (4–20)	—0.13	<0.01	2144.79	—0.12	<0.01	2140.38	0.02
School Social capital (7–34)	—0.13	<0.01		—0.13	<0.01		
Peer social capital (4–28)	—0.01	0.20		—0.01	0.58		
**Life satisfaction**							
Family Social Capital (4–20)	0.21	<0.01	1901.37	0.20	<0.01	1904.63	0.43
School Social capital (7–34)	0.12	<0.01		0.12	<0.01		
Peer social capital (4–28)	0.02	<0.01		0.02	0.33		
**15-year olds**							
**Health Complaints**							
Family Social Capital (4–20)	—0.12	<0.01	3024.42	—0.11	<0.01	3024.69	0.10
School Social capital (7–34)	—0.13	<0.01		—0.13	<0.01		
Peer social capital (4–28)	—0.01	0.14		—0.01	0.66		
**Life satisfaction**							
Family Social Capital (4–20)	0.16	<0.01	2318.96	0.14	<0.01	2316.34	0.03
School Social capital (7–34)	0.09	<0.01		0.09	<0.01		
Peer social capital (4–28)	0.04	<0.01		0.06	<0.01		

^a^ = Model include latent profiles as dummy variables.

**β** = Unstandardized beta coefficient.

AIC = Akaike’s Information Criteria, lower values indicate a better model fit

F-test = values below 0.05 indicate a better model fit (p = <0.05)

All models adjusted for gender and age, significant results is assumed at p<0.05.

## Discussion

In the present study we used a novel approach by applying Latent Profile Analysis to social capital research focusing on early to mid-adolescents. From a representative sample of Swedish adolescents this study identified profiles based on social capital in the contexts of family, school and peers. Overall, our results showed a positive distribution of social capital among adolescents, which given the representativeness of the sample is generalizable to the national Swedish context. In addition, our analysis uncovered a series of homogenous (in terms of social capital) groups that show how social capital co-occurs in an adolescent population. By looking at health outcomes in the identified profiles and comparing them, we were able to show the typical occurrence of frequent health complaints and level of life satisfaction that these profiles are likely to carry.

The ‘Low School’ and ‘Low Peer’ profiles found among 11-year olds represent opposites in social capital levels, showing that peer social capital exists independently from school social capital when the constructs of trust and sense of belonging are assessed. This profile was also found among 13- and 15-year olds. Arguably the ‘Low Peer’ profile illuminates individuals with negatively loaded peer relations outside the classroom or among specific classmates that does not affect their complete sense of trust and belonging in the school context. Observing the ‘Low School’ (11-year olds), ‘Low Fam&School’ (13-year olds) and the ‘Low Family’ (15-year olds) profiles that shared strong peer social capital, lead to the possible interpretation that adolescents with low trust and sense of belonging in the family and in school are more prone to seek strong social networks among peers outside school. However, it should not be assumed that those who gain social capital from peers are proffered with some protective factor effect against severe health outcomes. As has been shown previously, strong peer networks potentially entail what is known as ‘behavioural contagion’, which may instead exacerbate risk behaviours and consequently also affect health negatively [48]. Thus, the fact that a linear relationship of peer social capital with health could not be supported in this study is perhaps a sign of the duality of strong peer networks.

Other research has already dug deeper into social relations in adolescence, highlighting that peer-relationships may contribute to psychosocial development in different ways than the parent-child relationship [49]. Thus, peer relations can play an important role in shaping the social identity of adolescents [9] and has been linked to other health outcomes and to various risk behaviours [39]. In many domains the influence of parents is replaced by peer influence generally throughout adolescence [1, 50]. However, that strong trust and sense of belonging among peers would make up for poor social capital in the family in terms of frequent health complaints and life satisfaction could not be supported by this study.

There are some profiles that raise concern regarding health outcomes. First, the ‘All Low’ profile among 11-year olds averaged almost three health complaints at least twice a week. Such a frequency has been reported to place a burden on the individual that combined with emotional and psychological stress is likely to impair the everyday functioning [44], made visible also by the poor ratings of life satisfaction in this profile. With this in mind, the ‘Low Fam&School’ (13-year olds) and the ‘All Low’ profile (15-year olds) both averaged more than four health complaints at least twice a week, followed by life satisfaction scores of around four. Individuals with social capital levels fitting either of these profiles should be considered in high risk of severe health problems. In addition to these results, there are some results from our study that were a bit surprising. Among 11- and 15-year olds, the ‘Low School’ and ‘Low Family’ profiles were not significantly different from the ‘All Low’ profiles in terms of health outcomes despite stronger trust and sense of belonging in either the family or in school and strong peer relations. Besides again showing the peer context not to be able to work as a protective factor for poor health outcomes, these two profiles highlight adolescents that perhaps are harder to identify as in risk of severe health outcomes. Apart from having a not alarmingly low level of trust and sense of belonging in the family or in the school context, having a strong peer network may affect their willingness to seek help as previous research has shown [51].

Regarding the interaction between social capital in the family and in school, a link was visible when looking at the profiles, which has been described in previous research. This research shows that parental involvement in school, or lack of the same may be the factor that supports this link and possibly has an impact on student-teacher connectedness [52]. Parental involvement can enhance a student’s sociability, an important prerequisite supporting their ability to form social networks that build social capital [53]. It has also been suggested that children who have strong social capital in one context also are fortunate in other contexts [54], which in turn would infer the opposite when social capital is poor in one context. In light of the present findings, the above reasoning is perhaps more true regarding the contexts of family and school social capital, than of the peer context. The fact that no profile was characterized by diametrically opposite scores in family and school social capital can be interpreted as an indication of the link between family and school social capital. Despite that no such profile was discovered, reasonably there exist adolescents with poor trust and sense of belonging in the family that have strong social capital in school or vice versa. In this study, these individuals are likely to belong to the ‘Low School’ profile (11-year olds) or the ‘Low Family’ profile (15-year olds). Observation of the health outcomes in these profiles provides little indication that school social capital is able to compensate for poor social capital in the family or vice versa. There are a multitude of studies that suggest family social capital to be the most important context in relation to adolescent health [10, 36, 54]. The results of the regression analyses in this study concur with family social capital to be more strongly associated with life satisfaction. However, school social capital was equally able to predict health complaints. Further research is needed to better understand the interaction between family and school social capital in relation to health.

### Socioeconomic status and gender differences

There is existing evidence of an association between socioeconomic status and social capital, where strong social capital has been shown to buffer some of the negative consequences of low socioeconomic status on health [9]. In our study, profiles were investigated for differences in SES and how SES affected the predictions of health outcomes. Findings show that profiles were not different from each other in SES-levels, except from the ‘>Average’ profile in 13- and 15-year olds that was significantly higher than other profiles. Future research may direct more focus to the buffering effect of social capital on socioeconomic status among adolescents, perhaps to include measures of socioeconomic status that are reported by parents for higher reliability. In this study we found some interesting differences between profiles regarding gender distribution. Boys were in majority in all profiles with below average levels of social capital except for the ‘Low Fam&School’ and the ‘Low Family’ profile. This is a bit surprising given the known association between social capital and health outcomes, and the fact that girls generally report more health complaints than boys through adolescence, which would imply girls to be in majority in profiles with low social capital. The initial analyses that investigated gender differences in the total sample in each age group, found inconsistent differences between boys and girls in family and school social capital, but clearly showed that peer social capital was generally stronger among girls. Other research exploring gender differences in social capital also produces mixed findings. Some support a stronger relationship with health for girls [55] and some inconclusive differences [56]. Research in related fields, such as on social behaviour among adolescents help us to better interpret these gender differences. Prosocial behaviour for example, often seen as a facilitator for being able to benefit from social capital [57], has been found to develop earlier among girls than boys, however as boys’ prosocial behaviour increases in mid-adolescence, the gap starts to close [58]. Thus, prosocial behaviour may perhaps help explain why girls were in majority in the profiles of high social capital in all contexts. A more nuanced reasoning is needed when trying to understand the ‘Low Fam&School’ and ‘Low Family’ profiles that had a clear majority of girls. The work of Hombrados-Mendieta and colleagues [50] shows that girls turn to close friends for emotional support to a greater extent than boys in adolescence. This could explain why the profiles mentioned here shared above average scores in peer social capital, and had a majority of girls. The same gender distribution was not seen in the ‘Low School’ profile among 11-year olds. This may be partly because girls in early adolescence still rely on family for emotional support rather than on friends [50].

### Similarities and dissimilarities between age groups

To our knowledge, this study is one of few to investigate whether social capital patterns, within the contexts of family, school and among peers are differentiated between younger and older adolescents, While similarities were evident, unique profiles are important to highlight since it raises questions regarding the transformation of social networks during the early phase of adolescence. The ‘Low School’ (11-year olds), ‘Low Fam&School’ (13-year olds) and the ‘Low Family’ (15-year olds) profiles were those that had the most unique features, however common to all of them was they displayed strong peer social capital. This study also found an age difference when testing for a class clustering effect (seen only for the 11-year olds). This could indicate that younger adolescents are more ‘nested’, meaning their social networks are less likely to extend beyond family and classmates and values within those networks are shared exclusively within the group. This appears to suggest that age is of importance when investigating social capital among adolescents, a finding that might not have been considered previously. However, research by Collins and colleagues [49] on autonomy and self-regulation during early adolescence testifies to these age differences. They describe physical and psychological changes on-going during the pubertal transition that involve new stressors for the adolescent with consequential discrepant expectations in behaviour, actions and responses, between parent and adolescent [49]. This is explained as a part of a normative change in social behaviour and interaction that include boosted autonomy and increased peer influence. While such a pattern could only partially be supported by our profiles, the initial analyses reveal older adolescents to generally score slightly lower on family social capital. To further investigate changes in adolescents’ social behaviour and social capital, we encourage future research of longitudinal research design to address this issue.

### Implications

In summary, this study identified profiles that can help us understand more about how social capital in different contexts co-occur and how this translates into differences in health outcomes. Exploring ways through which social capital can be used for improved identification of vulnerable adolescents is of great interest. By itself, social capital may not be sufficient to further explain the pathways of health inequalities. However, identifying social capital profiles in a population of adolescents, representative for a national context to which we can add already existing knowledge on meso- and macrolevel structure, may aid us in our search for answers. This study is meaningful in the sense that it provides a rich image of social capital among adolescents and elucidates the interplay between multiple contexts and what it means in terms of health outcomes. Thus, LPA has the potential to offer insights complementary to regression analysis and other statistical techniques and, given the explorative design of the study, we encourage other researchers to undertake studies of similar design to further investigate the applicability of LPA in research on social capital and health among adolescents in other country contexts.

### Limitations

There are some methodological considerations that need to be discussed. First, the sample in this study is described as representative for Swedish adolescents. About 69 per cent of students who were eligible for participation in the selected classes, participated in the Swedish HBSC 2013/14 survey. There are various possible reasons why students would not have participated, such as absence from school that day, parents not wanting their child to participate or students themselves choosing not to participate. Since there were no data gathered that allowed us to evaluate the missing respondents being missing-at-random (MAR) we have to be aware of the possibility that the representativeness of the sample may be affected by missing data. Second, the cross-sectional nature of the data limits us to draw any conclusions about causal inference. We acknowledge previous critiques of research that explore the association between social capital and health, one point being to draw conclusions about the direction of this association without statistical evidence of such [59]. However, whilst our study did explore the relationships between different social capital profiles and health, the emphasis of the analysis was to uncover groups that have been invisible until now. Third, social capital has been measured and defined with great variation over the past decades [60], which can be problematic since using simply the phrase ‘social capital’ can mean different things depending on field of research. In this study we chose to include measures reflecting social cohesion, namely trust and sense of belonging within the family, in school and among peers. The items that were chosen are part of the HBSC-questionnaire that in total covers multiple dimensions of health and health behaviours (e.g. nutrition, physical activity, sedentary behaviour). Thus, the sole focus of the questionnaire is not on social capital. Nevertheless, these items have been validated for, and with adolescents, which speaks in their favour. Lastly, we want to make two clarifications concerning our statistical analysis. As mentioned, LPA provides multiple assessments of model fit considerations, which assists the researcher in choosing the ideal number of latent profiles [46]. This helps to minimize arbitrary decisions regarding the optimal model. Nonetheless, theoretical meaningfulness derived from a reality-connected understanding of the data should be embedded in the decision. Also, profiles identified through latent profile analysis have a degree of class probability, thus profiles are not ‘true’ but individuals instead have a high probability of belonging to this group while unlikely to belong to another depending on their social capital scores. When treating latent profiles as observed variables in subsequent analysis to predict distal outcomes, such as health complaints and life satisfaction, any uncertainty in individuals’ true class membership is ignored and as is the maximum posterior-probability rule. Combined with the limitations of imputing data under a model that is more restrictive than the latent profile analysis model, a risk of bias and attenuated results occurs [61]. This might be a contributing factor as to why our profiles were unable to add information to some of the multiple regression analysis.

## Conclusions

This study contributes new knowledge on how a person-centred approach to social capital can be beneficial for the identification of adolescents at risk of poor health outcomes. The profiles identified provide novel insights into how family, school and peer social capital generally co-occur in an adolescent population. These complex interactions here embodied as profiles give us a chance to examine the different layers of social capital and how they translate into differences in adolescent health outcomes. We conclude that the application of multiple analytical approaches for studying the role of social capital for adolescents’ health outcomes has the potential to improve translation of research into policy and practice for reduction of health inequalities amongst adolescents.
